# Positive emotional well-being and glucose control in a nationwide sample

**DOI:** 10.1007/s10865-025-00588-5

**Published:** 2025-08-14

**Authors:** Yasmin Shemali, Tasneem Khambaty, Zachary Goodman, Gail Ironson

**Affiliations:** 1 Department of Psychology, University of Miami, Miami, USA; 2 Department of Psychology, University of Maryland, Baltimore County, Baltimore, USA; 3 Department of Psychiatry, San Diego School of Medicine, University of California, La Jolla, USA

**Keywords:** Positive affect, Life satisfaction, Emotional well-being, HbA1c, Glucose control, Diabetes

## Abstract

To investigate the link between HbA1c, a marker of glucose control, and positive emotional well-being (PEWB). Data were from a nationwide survey (*N* = 1933) which included an older, chronically ill subgroup (*N* = 905). Two aspects of PEWB were assessed via cross-sectional regression analyses predicting HbA1c from positive affect and life satisfaction individually, controlling for demographic variables. HbA1c was analyzed via blood-spot from a finger-prick. The mediating role of health behaviors (smoking, alcohol, BMI, and moderate exercise) were also examined. Higher positive affect and life satisfaction were significantly related to lower HbA1c in the overall and older, chronically ill samples controlling for demographics, as well as health behaviors and depression. Individuals with lower positive affect and lower life satisfaction were at increased odds of having clinically elevated HbA1c (> 6.5), indicative of diabetes, in both the overall sample (OR = 1.37; and OR = 1.13) and the chronically ill, older sample (OR = 1.59; and OR = 1.14). Two health behaviors emerged as mediators in the overall sample: BMI and exercise. These findings suggest that PEWB factors such as positive affect and life satisfaction are associated with HbA1c in both the general population and older, chronically ill individuals. Health factors such as BMI and moderate exercise mediate this relationship.

## Introduction

Hyperglycemia, or elevated circulating glucose levels, is pervasive in American society. A hallmark of both Type 1 diabetes (T1DM) and Type 2 diabetes (T2DM), hyperglycemia is present in 9.4% of the U.S. population, or 30.3 million people (Centers for Disease Control and Prevention [CDC], 2017a, b). Glycosylated hemoglobin (HbA1c) is the primary measure of hyperglycemia and is considered a stable estimate as it reflects the average blood glucose concentration over the last 90–120 days. Thus, it has an advantage over other measures of glucose control such as fasting glucose, which only measures a single point in time. In turn, chronic hyperglycemia is linked to serious complications, including heart disease, blindness, stroke, and kidney disease (CDC, 2022). Although this paper focuses on HbA1c and not specifically T2DM, it has clinical relevance for T2DM, therefore we include discussion of studies among individuals with T2DM as well as the implications of our findings in the context of T2DM.

An extensive literature has focused on the relationship between negative psychological states (e.g., depression, anxiety) and deleterious outcomes in T1DM or T2DM. Case in point, the literature has established a bi-directional association between depression and T2DM outcomes. Meta-analytic evidence indicates that depression is an independent risk factor for T2DM, with risk ratios similar to traditional T2DM risk factors (Pratt & Brody, 2008). In turn, adults with T2DM are twice as likely to exhibit depressive symptoms as adults without this condition (Anderson et al., 2001). These negative psychological states in T2DM warrant investigation because they are readily amenable to psychosocial interventions, and treatment can vastly improve patients’ abilities to cope with the burden of diabetes self-management, improve glycemic control, prevent future diabetes complications.

Simultaneously, a smaller literature has emerged on positive psychological states such as joy, happiness, and contentment, and their association with various health outcomes (Seligman, 2008). This emerging literature is timely because delineating the impact of positive psychological states in conjunction with negative states on health outcomes can better inform the development of interventions aimed at reducing disease burden. Studies in this literature have indicated that positive psychological states are linked to favorable health outcomes in several disease conditions, including coronary disease, stroke, and HIV (Pressman & Cohen, 2005; Ironson & Hayward, 2008). They are also associated with overall mortality in both the general population and individuals with health conditions (Chida & Steptoe, 2008; Ironson et al., 2020).

A few studies have shown a significant, negative relationship between positive psychological factors and blood glucose levels, including positive affect (Tsenkova et al., 2008) and purpose in life (Boylan et al., 2017). Favorable outcomes have also been reported in the context of T2DM (Celano et al., 2013). For instance, women with higher existential well-being report better glycemic control (Newlin et al., 2007, 2008), with higher social support and better T2DM management considered possible mediators of this link (Brody et al., 2008). Higher levels of life satisfaction and emotional vitality (Boehm et al., 2015) and psychological well-being (Sun et al., 2022) have been associated with reduced odds of self-reporting a physician diagnosis of diabetes. Furthermore, in individuals with parental history of diabetes, research suggests positive affect may buffer an individual’s risk for developing T2DM (Tsenkova et al., 2016). Links to greater health behavior adherence and lower mortality have also been demonstrated (Rose et al., 2002; Yi et al., 2008; Al-Khalwaldeh et al., 2012; Nakahara et al., 2006; Moskowitz et al., 2008).

Within this literature on positive psychological states, positive emotional well-being (PEWB) is defined as the presence of high positive affect and life satisfaction, in the absence of depression (Lopez et al., 2018). Positive affect specifically includes emotions that reflect the level of pleasurable engagement in one’s environment, while life satisfaction refers to a cognitive, judgmental orientation that encompasses a person’s view of their quality of life. (Diener et al., 1985; Clark et al., 1989). We chose to focus on PEWB in this paper as it is a well-established theoretical construct that includes measures of both the affective and cognitive components of well-being (Lopez et al., 2018).

Only a small literature exists examining the relationship between positive affect and HbA1c, and even fewer articles on life satisfaction and HbA1c, despite the importance of HbA1c for diabetes and health. Moreover, there is a need to investigate the mechanisms underlying any glycemic benefits of PEWB, such as through health behaviors. This study follows prior work published by our group exploring the role of gratitude in HbA1c (Krause et al., 2017), and the link between mood and diabetes risk (Khambaty et al., 2022), while further expanding the research agenda on positive psychological states and a biomarker most relevant for diabetes, but also relevant for metabolic syndrome and cardiovascular disease (Dilley et al., 2007; Khaw et al., 2004; Kim et al., 2008).

Consequently, the primary objective of this study was to examine the association between PEWB and glycemic control as assessed by HbA1c. We examined the individual roles of positive affect and life satisfaction with HbA1c above depression in (a) a large community sample from the Landmark Spirituality and Health Survey (LSHS), and (b) a subgroup of older individuals from the LSHS with at least one chronic disease, to better understand the relationship between PEWB and HbA1c. We examined the individual associations of positive affect and life satisfaction with HbA1c, rather than their combined associations, with the aim of parsing apart their individual roles in the relationship. In addition, they are conceptually different, as positive affect relates more strongly to current emotion while life satisfaction has a stronger cognitive component with a longer timeframe. We chose to examine the subgroup of older adults with at least one chronic illness because hyperglycemia is more prevalent in older adults and those with chronic illnesses, allowing us to investigate the role of PEWB in individuals with higher risk for hyperglycemia. We hypothesized that higher positive affect and life satisfaction would both be related to lower HbA1c in both the overall and older, chronically ill subsample.

Our secondary objective was to calculate odds ratios based on clinical cut-offs for HbA1c levels (e.g., normal, pre-diabetes, and diabetes) to contribute clinically relevant implications of our findings. We hypothesized that in both the overall sample and the older, chronically ill sample, that individuals with high levels of PEWB would be significantly less likely to have HbA1c levels in the pre-diabetic or diabetic range, while individuals with low levels of PEWB would be significantly more likely to have elevated HbA1c levels.

Given the established links between HbA1c and health behaviors (Cavero-Redondo et al., 2018; Vlassopoulos et al., 2013), and between PEWB and health behaviors such as greater physical activity, lesser alcohol use, not smoking, and better diet (Conry et al., 2011; Lappan et al., 2020; Hogan et al., 2015; Boehm et al., 2018), our third objective was to examine whether associations between HbA1c and PEWB were mediated by four health behaviors: smoking, alcohol use, moderate exercise, and BMI. In line with Fredrickson’s (2001) Broaden-and-Build Theory, PEWB may reduce the likelihood of alcohol, tobacco, and substance use by enhancing creative problem-solving and strengthening social support networks (Van Cappellen et al., 2018). Finally, higher BMI and obesity have been associated with less frequent positive affect (Carr et al., 2007), and positive emotions have been shown to predict weight loss intentions and behaviors (Richards et al., 2021). There is also evidence that a healthy weight improves life satisfaction (Habibov et al., 2019).

## Methods

This study was conducted as part of the Landmark Spirituality and Health Survey (LSHS). LSHS was administered in 2014 to a representative sample of 3010 adults in the United States. Data were collected through in-person interviews by the National Opinion Research Center (NORC). Clustered random household sampling was employed on 44 national frame areas which represented the continental United States, with a response rate of 50%. NORC’s IRB approved this study. Participants were compensated $25. See the LSHS website for more information regarding the survey (http://landmarkspirituality.sph.umich.edu/).

The overall sample in the present study was made up of 1933 participants for whom the biomarker HbA1c was collected. The average age of the overall sample was 52.12, SD = 19.24 (range 17 to 96) years. There were more females (58%) than males (42%), and the mean years of education was 13.41 (SD = 3.21). The sample was 68% white, 12.7% Black, 15.9% Hispanic, and 1.9% Asian. The older, chronically ill subgroup referenced in this paper consisted of 905 individuals who were 50 years or older and reported at least one current chronic disease. See Table 1 for means and standard deviations of demographics and key study variables for both groups.

**Table 1:** Descriptive statistics for key variables

	Overall sample with HbA1c (*n* = 1933)	Chronic illness & Age ≥ 50 (*n* = 905)	Non-chronic illness & Age < 50 (*n =* 467*)*
Age *M (SD)*	52.12 (19.24)	67.03 (10.06)**	37.82 (13.49)
Sex (male)	42.0%	41.1%	43.8%
Black	12.7%	10.9%*	14.3%
Asian	1.9%	1.1%	2.7%
Hispanic	15.9%	9.5%**	21.5%
Education *M (SD)*	13.41 (3.21)	13.34 (3.37)**	13.55 (3.03)
HbA1c			
Median	5.44	5.72	5.17
25th– 75th % range	5.16–5.85	5.30–6.13	4.89–5.58
>5.7 (%) (prediabetic)	38.6	53.8	15.0
>6.5 (%) (diabetic)	12.0	17.6	3.2
Positive affect *M (SD)*	18.28 (3.67)	18.39 (3.68)	18.35 (3.65)
Life satisfaction *M (SD)*	10.74 (2.63)	10.91 (2.64)	10.65 (2.55)
Depression *M (SD)*	6.07 (2.59)	5.92 (2.50)	6.10 (2.61)
BMI *M (SD)*	20.03 (6.59)	29.35 (6.30)	28.44 (6.65)
Smoke	22.8%	17.6%**	27.4%
Alcohol (drinks/month) *M (SD)*	14.47 (34.03)	12.14 (28.77)**	17.01 (37.37)
Exercise (days/week) *M (SD)*	3.62 (2.55)	3.56 (2.63)**	3.74 (2.44)

χ^2^ test used for categorical variables; t-tests used for continuous variables

*Statistically significant difference in means (vs. non-chronic illness) at the *p* < 0.05 level. **p < 0.01 level

### Measures

#### Positive affect

Positive affect was evaluated through a subset of five items from the PANAS (Watson et al., 1988). Respondents indicated, on a Likert scale of one to five, the extent to which they had felt inspired, alert, determined, attentive, and active in the past month. Higher scores reflected higher positive affect.

Ratings on each item were summed together to calculate the total score. Scores ranged from five to 25, with higher scores indicating higher positive affect (alpha = 0.78).

#### Life satisfaction

Life satisfaction was assessed using a subset of three items from Diener’s (1985) five-item Satisfaction with Life Scale. This abbreviated, three-item version of the SWLS, known as the SWLS-3 has demonstrated comparable reliability and validity to the 5-item scale, including high internal consistency, test-retest reliability, measurement invariance, and convergent validity (Kjell & Diener, 2021). Respondents expressed on a five-point Likert scale how much they agreed or disagreed with three statements: “the conditions of my life are excellent”; “I am satisfied with my life”; “in most ways my life is close to my ideal.” Ratings were summed together for a total score, which ranged from three to 15; higher scores reflected greater life satisfaction. The scale had good internal consistency (alpha = 0.85).

#### Depressive symptoms

The Center for Epidemiologic Studies-Depression Scale (CES-D) (Radloff, 1977) was used to measure depressive symptoms. Respondents reported the frequency with which they had experienced four affective depressive symptoms: “I felt I could not shake off the blues, even with the help of my family and friends,” “I felt depressed,” I had crying spells,” and “I felt sad.” The four items of the CES-D scale that measured somatic symptoms of depression were not used as they could have been due to chronic illness rather than depression. Respondents rated frequency using a four-point Likert scale ranging from Rarely/None of the time (1) to Most/All of the time (4). Their responses were summed to create a total score; higher scores indicated greater depressive symptoms. Reliability was good (alpha = 0.85).

#### Chronic health conditions

Respondents were given a list of 12 chronic illnesses and asked to indicate which, if any, they had experienced during the last 12 months. The list of chronic illnesses consisted of arthritis/rheumatism, cataract/glaucoma/eye disease, asthma/ emphysema/other respiratory diseases, hypertension, heart attack/ issues, diabetes, ulcers, liver disease, kidney disease, urinary tract disorder, cancer/malignant tumor, and prostate or other health condition) (Liang, 1990). Those who endorsed at least one chronic illness, and who were 50 years or older, were included in the older, chronically ill subgroup analyses.

#### Health behavior variables

Four health-related variables– Body Mass Index (BMI), smoking, alcohol use, and moderate exercise– were included in the analysis as both control variables and possible mediators. BMI is often used in the medical setting to evaluate whether a person meets criteria for overweight or obesity. It was calculated from individuals’ measured height and weight. Although BMI is usually deemed a medical control variable and is not a health behavior, we also considered it as a possible mediator since it can be affected by health behaviors such as diet and weight loss. Alcohol use was defined by the number of alcohol drinks an individual consumed each month. Smoking was defined as whether the participant currently smokes (1 = yes, 0 = no). Moderate exercise was operationalized as the number of days per week in which the individual performed 15 minutes or more of moderate-intensity exercise.

#### Demographic control variables

Sex, education, race/ethnicity, age, and BMI were utilized as control variables. Among these, age and BMI represent biological control variables that are strongly linked to HbA1c levels. Sex was a dummy variable (1 = male; 0 = female), and race/ethnicity was coded as (1 = African American, 0 = other). Research consistently indicates that African Americans have higher HbA1c levels, in both samples of individuals with and without T2DM (Kirk et al., 2006); thus, we controlled for this variable to account for this difference in risk of hyperglycemia.

#### HbA1c

At the end of the interviews, a blood sample was collected (from those who agreed; *N* = 1933) via a capillary finger stick with a disposable lancet. Between three and five blood spots were applied to filter paper and shipped to the Department of Laboratory Medicine at the University of Washington for analysis. The HbA1c Assay used to measure glycosylated hemoglobin (HbA1c) in dried blood spot (DBS) samples is similar to a previously published method (Egier et al., 2011). The assay utilized an automated ion-exchange high-performance liquid chromatography (IE-HPLC) system (Variant II Hemoglobin Testing System, Bio-Rad). A detailed discussion of how the HbA1c assays were conducted is provided in the supplemental materials that are attached to this report. Outliers were Winsorized (Ratcliff, 1993), such that HbA1c scores that fell beyond three standard deviations above the mean were replaced with scores that fell exactly at three standard deviations above the mean. This makes it possible to deal with the unwanted influences of outliers while retaining highly skewed cases so that the sample remains representative. We decided against using a full transformation to adjust for skew because it might have affected the clinical interpretation of the data. The raw score range for HbA1c values was 3.96–16.48. However, after using the Winsorizing procedure, the range was 3.96–8.13 (see Table 1).

### Data analyses

A four-step data analysis procedure was employed using SPSS software version 22 in order to probe the relationship between each PEWB variable (i.e., positive affect or life satisfaction), HbA1c, and covariates. Thus, each model was employed twice– once for positive affect and once for life satisfaction– with the PEWB variables being run separately. Model 1 investigated the association between each PEWB variable and HbA1c using hierarchical linear regression and controlling for demographics (age, sex, race/ethnicity, and education). In model 2, depression was added as a covariate. This allowed us to test whether the PEWB variables would still predict HbA1c, over and above depression. In model 3, depression was removed from the model, and health behaviors (BMI, smoking, alcohol use, and moderate exercise) were added as covariates so that we could determine whether PEWB would still predict HbA1c over and above health behaviors. Models 2 and 3 were thus designed to disentangle depression and health behaviors as contributors to HbA1c. Finally, in model 4, depression, health behaviors, and demographics were covariates, with the aim of determining whether PEWB would still predict HbA1c with both depression and health behaviors taken into account. All analyses were conducted without replacement for missing data given the small proportion of missing data (< 1.2% for demographics; <1.3% for psychological variables; <1% for health behavior variables, 3.8% for BMI). In addition, while we used two-tailed tests for all our analyses, we did report one-tailed findings given our directional hypotheses that higher PEWB would be related to lower HbA1c.

Next, in order to facilitate clinical interpretations of our results, HbA1c levels were categorized based on clinical cut-offs for pre-diabetes and diabetes established by the American Diabetes Association (2010) (i.e., HbA1c *≥* 5.7 is classified as pre-diabetic; HbA1c *≥* 6.5 is diabetic). Individuals were also categorized as “high,” “moderate,” or “low” on positive affect and life satisfaction based on their scores on each of the PEWB measures. Positive affect and life satisfaction were divided into tertiles for this purpose. Using logistic regression, odds ratios for having pre-diabetes and diabetes were then calculated in relation to having high, moderate or low levels of positive affect and life satisfaction. High levels of positive affect and life satisfaction were used as the reference categories in these analyses.

Finally, tests of mediation were conducted to determine the indirect effects of the two PEWB variables on HbA1c through health behaviors. These analyses were conducted using the PROCESS macro for SPSS (Hayes, 2012) and Mplus 8.8. The PROCESS macro was used to test the indirect effects for the continuous health behavior variables: BMI, alcohol use, and moderate exercise. Because the smoking variable was a dichotomous measure, it was not possible to model the proposed indirect effect through the PROCESS macro in SPSS. Consequently, we tested this effect through Mplus using Bayesian estimation and specifying the mediator as a continuous latent variable represented by the dichotomous smoking measure (Muthén, 2011).

## Results

Table 1 provides descriptive statistics (i.e., means and standard deviations) for HbA1c levels, psychological variables, and health behaviors for the overall sample and the chronically ill, older adult subgroup. This latter group had a lower percentage of Asian and Hispanic participants and a higher percentage of Black participants than the overall sample. In comparison to their younger, healthy counterparts, the chronically ill, older subgroup had significantly lower levels of education, exercise, and alcohol consumption. They also had higher average BMI and were less likely to smoke.

### Correlations among key variables

Table 2 illustrates correlations between key study variables in the overall sample. Those with higher HbA1c were significantly more likely to be of an older age, male gender, and have less education. African Americans were more likely to have higher HbA1c (*r* = 0.084, *p* < 0.001; not shown) than non-African Americans. Higher HbA1c was also significantly associated with higher BMI, non-smoking, less alcohol consumption, less days of moderate exercise, and lower levels of positive affect. Higher positive affect and life satisfaction were both associated with lower BMI, non-smoking, greater exercise, and lower depression. However, only life satisfaction was significantly associated with older age. Life satisfaction and positive affect were moderately correlated with one another (*r* = 0.27, *p* < 0.001).

**Table 2:** Bivariate correlations among key variables

	1	2	3	4	5	6	7	8	9	10
1 HbA1c										
2 Age	0.31**									
3 Sex^a^	0.07**	− 0.03								
4 Education	− 0.09**	0.003	0.016							
5 BMI	0.23**	0.04	− 0.045*	− 0.04						
6 Smoking	− 0.07**	− 0.18**	0.07**	− 0.19**	− 0.07**					
7 Alcohol	− 0.09**	− 0.08**	0.20**	0.02	− 0.08**	0.18**				
8 Moderate exercise	− 0.08**	− 0.03	0.08**	0.11**	− 0.17**	− 0.03	0.06**			
9 Depression	0.03	− 0.06**	− 0.11**	− 0.16**	0.04*	0.13**	0.02	− 0.14**		
10 Positive affect	− 0.08**	-0.01	− 0.03	0.21**	− 0.05**	− 0.05**	0.01	0.28**	− 0.28**	
11 Life satisfaction	− 0.03	0.07**	0.02	0.08**	− 0.06**	− 0.17**	− 0.03	0.15**	− 0.44**	0.27**

HbA1c = hemoglobin A1c; BMI = body mass index; ^a^Male=1; Female = 0

*p < 0.05. **p < 0.01

### HbA1c regressed on positive affect

Linear regression analysis was used to examine the relationship between positive affect and HbA1c (see Table 3). Positive affect was significantly related to lower HbA1c in both the overall sample (Model 1: b = -0.070, t = -3.168, *p* = 0.002) and the chronically ill, older subgroup (Model 1: b = -0.109, t = 3.176, *p* = 0.002) when controlling for demographic variables (i.e., age, gender, race/ethnicity, education). Depression was significantly related to higher HbA1c before adding PANAS into the model in both the overall sample (Model 2: B = 0.049, t = 2.202, *p* = 0.028) and chronically ill, older sample (Model 2: B = 0.105, t = 3.090, *p* = 0.002, (Not in table). When depression was added into the model, positive affect remained significantly related to HbA1c in both the overall sample (Model 2: b = -0.062, t = 2.692, *p* = 0.007) and the chronically ill, older group (Model 2: b = -0.006, t = -2.393, *p* = 0.017). This significant, negative relationship remained after controlling for health behaviors smoking, alcohol consumption, moderate exercise, and BMI. This was true in both the overall sample (Model 3: b = -0.057, t = -2.458, *p* = 0.014) and the chronically ill, older subgroup (Model 3: b = -0.108, t = -3.005, *p* = 0.003). When all three covariate groups (demographics, depression, and health behaviors) were controlled, positive affect remained significantly related to lower HbA1c in the older, chronically ill sample, but was only significantly related to lower HbA1c in the overall sample on a one-tailed test.

**Table 3:** Hemoglobin A1c regressed on positive affect

	Model 1	Model 2	Model 3	Model 4
	Overall (chronic)	Overall (chronic)	Overall (chronic)	Overall (chronic)
Body mass index	-	-	0.206** (0.210**)	0.205** (0.209**)
Smoking	-	-	− 0.001 (0.003)	-0.006 (0.001)
Alcohol	-	-	− 0.065** (-0.128**)	− 0.064** (-0.124**)
Moderate exercise	-	-	− 0.017 (0.001)	− 0.016 (0.007)
Depression	-	0.031 (0.079*)	-	0.038 (0.082*)
Positive affect	− 0.070** (-0.109**)	− 0.062** (-0.086*)	− 0.057* (-0.108**)	− 0.047† (-0.086*)

Standardized regression coefficients are presented for both the overall sample (and the chronically ill, older adult group). Chronic was defined as having ≥ 1 chronic illness. Older adults were defined as ≥ 50 years old. Model 1: adjusted for age, sex, race/ethnicity, and education. Model 2: adjusted Model 1 variables plus depression. Model 3: adjusted for Model 1 plus smoking, alcohol, moderate exercise, and body mass index. Model 4: adjusted for model 3 plus depression

^†^*p* < 0.10. *p < 0.05. **p < 0.01. Chronic was defined as having ≥ 1 chronic illness

### HbA1c regressed on life satisfaction

Table 4 presents the relationship between life satisfaction and HbA1c. Life satisfaction was significantly associated with lower HbA1c with demographic covariates in both the overall sample (Model 1: b = -0.063, t = -2.896, *p* = 0.004) and chronically ill, older group (Model 1: b = -0.095, t = -2.840, *p* = 0.005). Depression was significantly related to higher HbA1c before adding life satisfaction into the model in both the overall sample (Model 2: b = 0.007, t = 2.033, *p* = 0.042) and chronically ill, older sample (Model 2: b = 0.012, t = 2.873, *p* = 0.004). When depression was added into the model as a covariate, life satisfaction remained significant for the overall sample (Model 2: b = -0.055, t = -2.313, *p* = 0.021) but was only significant on the one-tailed test for the older, chronically ill group (Model 2: b = -0.071, t = -1.914, *p* = 0.056). Life satisfaction also maintained its significant, negative relationship with HbA1c after controlling for demographics and health behaviors in the overall sample (Model 3: b = -0.058, t = -2.616, *p* = 0.009) and the chronically ill, older group (Model 3: b = -0.103, t = -2.983, *p* = 0.003). Finally, when all three covariate groups (demographics, depression, and health behaviors) were controlled, life satisfaction remained significantly related to lower HbA1c in the older, chronically ill sample (Model 4: b = -0.077, t = -2.053, *p* = 0.040) but was only significantly related to lower HbA1c in the overall sample on a one-tailed test (Model 4: b = -0.047, t = -1.944 *p* = 0.052).

**Table 4:** Hemoglobin A1c regressed on life satisfaction

	Model 1	Model 2	Model 3	Model 4
	Overall (chronic)	Overall (chronic)	Overall (chronic)	Overall (chronic)
Body mass index	-	-	0.206** (0.207**)	0.205** (0.205**)
Smoking	-	-	− 0.01 (-0.007)	− 0.012 (-0.007)
Alcohol	-	-	− 0.064** (-0.128**)	− 0.064** (-0.124**)
Moderate exercise	-	-	− 0.022 (-0.003)	− 0.021 (0.002)
Depression	-	0.021 (0.068^†^)	-	0.029 (0.072^†^)
Life satisfaction	− 0.063** (-0.095**)	− 0.055* (-0.071^†^)	− 0.058** (-0.103**)	− 0.047^†^ (-0.077*)

Standardized regression coefficients are presented for both the overall sample (and the chronically ill, older adult group). Chronic was defined as having ≥ 1 chronic illness. Older adults were defined as ≥ 50 years old. Model 1: adjusted for age, sex, race/ethnicity, and education. Model 2: adjusted Model 1 variables plus depression. Model 3: adjusted for Model 1 plus smoking, alcohol, moderate exercise, and body mass index. Model 4: adjusted for model 3 plus depression

^†^*p* < 0.10. *p < 0.05. **p < 0.01. Chronic was defined as having ≥ 1 chronic illness

### Odds ratios for clinically elevated HbA1c

Table 5a presents odds ratios in the overall sample for having HbA1c between 5.7 and 6.4, which represents the pre-diabetes range. 35.3% of high positive affect individuals in the overall sample had an HbA1c level between 5.7 and 6.4, as compared with 41.6% of low positive affect individuals. Logistic regression was used to evaluate the odds ratios for being pre-diabetic, controlling for demographics. Individuals who were low on positive affect were significantly more likely (OR = 1.35) to have HbA1c greater than or equal to 5.7 (i.e., pre-diabetic range) than those who were high on positive affect. In other words, individuals with high levels of positive affect were less likely to be pre-diabetic than individuals with low levels of positive affect. The odds ratio findings for life satisfaction in the overall sample were contrasting with those of positive affect. Logistic regression controlling for demographic variables indicated that individuals with moderate levels of life satisfaction were significantly more likely (OR = 1.33) to have HbA1c in the pre-diabetic range as compared to those with high satisfaction. Those with low life satisfaction were more likely (OR = 1.25) than those with high satisfaction to have HbA1c in the pre-diabetic range, but this difference was not significant.

**Table 5:** Proportions and odds ratios for positive emotional well-being and HbA1c

	Proportion (%) with HbA1c levels > 5.7 and < 6.5 (prediabetic, not including diabetic)	Odds ratio^a^	95% confidence interval
*(a) In the pre-diabetic range in overall sample*			
Positive affect			
High	35.1	1	
Moderate	37.4	1.02	0.791, 1.316
Low	36.1	1.01	0.777, 1.325
Life satisfaction			
High	33.4	1	
Moderate	38.7	1.13	0.872, 1.471
Low	33.4	1.05	0.814, 1.367

	Proportion (%) with HbA1c levels > 6.5 (diabetic)	Odds ratio^a^	95% confidence interval
*In the diabetic range in overall sample*			
Positive affect			
High	9.9	1	
Moderate	13.7	1.48	1.12, 1.94
Low	13.7	1.37	1.03, 1.82
Life satisfaction			
High	11.5	1	
Moderate	13.5	1.20	0.92, 1.56
Low	12.1	1.13	0.86, 1.49

	Proportion (%) with HbA1c levels > 5.7 and < 6.5 (prediabetic, not including diabetic)	Odds ratio^a^	95% confidence interval
*(b) In the pre-diabetic range among chronically ill, older adults*			
Positive affect			
High	31.5	1	
Moderate	31.2	0.954	0.71, 1.28
Low	31.0	1.00	0.74, 1.35
Life satisfaction			
High	32.6	1	
Moderate	30.9	0.99	0.74, 1.36
Low	29.4	1.04	0.78, 1.40

	Proportion (%) with HbA1c levels > 6.5 (diabetic)	Odds ratio^a^	95% confidence interval
*In the diabetic range among chronically Ill, older adults*			
Positive affect			
High	13.8	1	
Moderate	18.9	1.44	1.11, 2.29
Low	21.2	1.59	1.01, 2.04
Life satisfaction			
High	17.2	1	
Moderate	19.4	1.04	0.74, 1.47
Low	17.1	1.14	0.81, 1.62

HbA1c = Hemoglobin A1c; ^a^Adjusted for age, sex, race/ethnicity, education

Table 5a also shows the odds ratios in the overall sample for having HbA1c greater than or equal to 6.5, which represents the diabetes range. 13.7% of individuals with moderate positive affect, and 13.7% of individuals with low positive affect, had HbA1c levels in the diabetic range (*≥* 6.5), as compared to 9.9% of individuals with high positive affect. Logistic regression controlling for demographic variables indicated that individuals with moderate and low positive affect were significantly more likely (OR = 1.48 and 1.37, respectively) to have HbA1c levels in the diabetic range when compared to those with high positive affect. 11.5% of individuals with high life satisfaction had A1C levels in the diabetic range, compared to 12.1% of individuals with low life satisfaction. Individuals with moderate and low life satisfaction were more likely to have HbA1c levels in the diabetic range (OR = 1.20 and 1.13 respectively), but these differences in odds were not significant.

Table 5b shows the odds ratios in the chronically ill, older adult group. 49.9% of individuals with high positive affect had HbA1c greater than or equal to 5.7 (pre-diabetic range), compared to 65.1% of individuals with low positive affect. In this subgroup of adults, logistic regression showed that individuals with low positive affect had significantly greater odds (OR = 1.77) of having HbA1c in the pre-diabetic range when compared to individuals with high positive affect. 54.5% of individuals with high life satisfaction had HbA1c in the pre-diabetic range, as compared to 60.3% and 57.6% in the moderate and low life satisfaction groups, respectively. The odds ratios for the moderate and low life satisfaction groups were 1.25 and 1.28 respectively; these differences in odds, however, were not statistically significant in comparison to individuals with high life satisfaction.

**Fig. 1:**
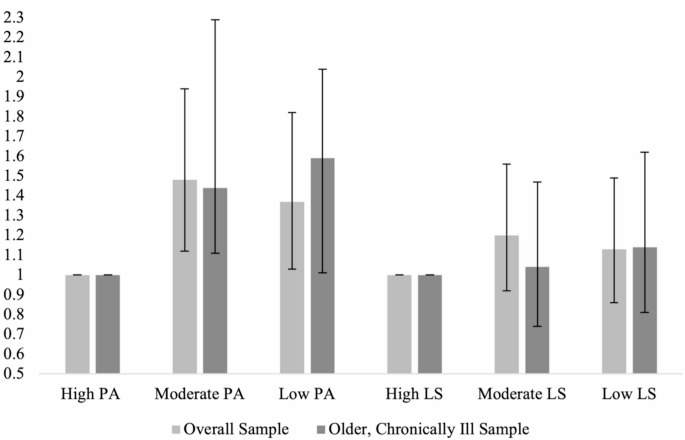
Odds ratio for HbA1c elevation into the diabetic range by PEWB level

Finally, Table 5b also presents the proportions and odds ratios of having HbA1c greater than or equal to 6.5 (i.e., diabetic range) for the chronically ill, older subgroup. 13.8% of those with high positive affect had HbA1c levels in the diabetic range, compared to 18.9% in the moderate positive affect group and 21.2 in the low positive affect group. Logistic regression indicated that individuals with moderate and low positive affect had a significantly greater odds of being in the diabetic group than individuals with high levels of positive affect (OR = 1.44 and 1.59, respectively). 17.2% of individuals with high life satisfaction had HbA1c levels in the diabetic range, compared to 19.4% of individuals with moderate life satisfaction and 17.1% of individuals with low life satisfaction. See Fig. 1 for a bar chart of odds ratios by PEWB level with their confidence intervals that summarizes the findings of these analyses.

### Mediation results

Correlations between the two PEWB variables (positive affect and life satisfaction) and each of the four health behaviors (smoking, alcohol use, moderate exercise, and BMI) were examined (see Table 2) before testing for the potential mediating role of health behaviors. In the overall sample, higher positive affect and life satisfaction were both significantly negatively related to BMI and smoking, and significantly positively related to moderate exercise. Neither positive affect nor life satisfaction was significantly correlated with alcohol use in the overall sample. The results of the mediation analysis are presented in Table 6. Standardized indirect effects, 95% confidence intervals, and the ratio of the indirect effect to the total effect are included. Indirect effects with a confidence interval that does not include zero are significant.

**Table 6:** Indirect effects of positive emotional well-being on HbA1c for health behaviors

	Overall sample				Chronically Ill, older group			
	Indirect effect^a^	Boot SE	95% CI	Ratio^b^	Indirect Effect^a^	Boot SE	95% CI	Ratio^b^
Positive affect								
BMI	− 0.016*	0.006	− 0.028, − 0.006	0.533	− 0.013	0.008	− 0.030, 0.003	0.325
Alcohol	− 0.001	0.003	− 0.006, 0.005	0.047	− 0.007	0.005	− 0.017 0.004	0.210
Exercise	− 0.013	0.007	− 0.027, 0.001	0.481	− 0.012	0.012	− 0.035, 0.011	0.336
Smoking	0.000	0.001	− 0.001, 0.001	0.000	− 0.013	0.022	− 0.034, 0.006	0.448
Life satisfaction								
BMI	− 0.010	0.005	− 0.020, 0.000	0.328	− 0.004	0.008	− 0.020, 0.012	0.102
Alcohol	0.002	0.002	− 0.003, 0.007	− 0.081	− 0.004	0.006	− 0.017, 0.006	0.118
Exercise	− 0.007*	0.004	− 0.014, − 0.001	0.275	− 0.009	0.008	− 0.025, 0.006	0.223
Smoking	0.012*	0.007	− 0.001, 0.026	0.200	0.017*	0.011	− 0.001, 0.041	0.189

Boot SE = bootstrapped standard error; CI = confidence interval.

^a^Standardized coefficients; ^b^Ratio of indirect effect to total effect. *p < 0.05.

Posterior standard deviation from Bayesian estimation

### Health behavior mediation for the overall sample

In order for variables to be considered as mediators, they need to be significantly related to the predictor as well as the outcome. Two health behaviors emerged as potential mediators of the relationships between PEWB and HbA1c in the overall sample. After testing, a significant, indirect effect of positive affect on HbA1c through BMI was observed; higher positive affect was related to lower HbA1c through BMI when controlling for demographic variables. This indirect effect constituted 53.3% of the total effect. Secondly, a significant, indirect effect of life satisfaction on HbA1c through exercise was also present. Thus, higher life satisfaction was related to lower HbA1c through moderate exercise. This indirect effect accounted for 27.5% of the total effect. There was a significant indirect effect of life satisfaction on HbA1c through smoking behavior, although the confidence interval to three decimal places included zero so we declined to interpret this.

### Health behavior mediation in the older, chronically Ill subgroup

Because the indirect effects in the older, chronically ill subgroup through alcohol, BMI, and moderate exercise were not significant, there was little evidence of the mediating role of the health behaviors in the relationship for positive affect and HbA1c or for life satisfaction and HbA1c. However, there was a significant indirect effect of life satisfaction on HbA1c through smoking behavior, although the confidence interval to three decimal places included zero so we declined to interpret this.

## Discussion

In the overall sample, individuals with lower positive affect and lower life satisfaction were at increased odds (OR = 1.37 and OR = 1.13) of having clinically elevated HbA1c (> 6.5), indicative of diabetes, controlling for demographics and health behaviors. When depression was added into the model, in the overall sample, both positive affect and life satisfaction remained significantly associated with HbA1c levels. Additionally, two health behaviors emerged as potential mediators in the overall sample: BMI for the relationship between positive affect and HbA1c, and exercise for the relationship between life satisfaction and HbA1c. In the chronically ill, older sample, individuals with lower positive affect and lower life satisfaction were also at increased odds (OR = 1.59 and OR = 1.14) of having clinically elevated HbA1c (> 6.5), indicative of diabetes, controlling for demographics and health behaviors. When depression was added into the model, positive affect remained significant, but life satisfaction was only significant on the one-tailed test. Positive affect and life satisfaction were not associated with significant differences in odds of being pre-diabetic (i.e., HbA1c levels > 5.7 and < 6.5) in either sample.

Our findings are consistent with the few other studies in the literature that have examined PEWB and glycemic control specifically. Among these is a large epidemiological study, which found that higher levels of emotional vitality and life satisfaction were associated prospectively with up to a 15% decrease in the odds of physician-diagnosed diabetes; positive affect was not examined in that study (Boehm et al., 2015). In another study of Black women with T2DM, higher existential well-being was associated with better glycemic control (Newlin et al., 2007, 2008). More generally, our findings are consistent with the literature demonstrating the beneficial effect of other subjective well-being constructs such as optimism, resilience, and self-efficacy on better glycemic control (Boehm et al., 2015; Rose et al., 2002; Yi et al., 2008; Al-Khawaldeh et al., 2012; Nakahara et al., 2006).

It is notable that even in the subgroup of participants who reported at least one chronic disease, positive affect was inversely linked to HbA1c perhaps suggesting better management of disease, and in the long term, perhaps increased longevity. Consistent with this hypothesis, in a large study including 700 participants with physician-diagnosed diabetes, those who reported higher levels of positive affect had a reduced risk of mortality across 10 years (Moskowitz et al., 2008). However, in contrast to our findings, this study reported that the protective effect of positive affect was attenuated when controlling for negative affect. More generally, meta-analytic findings in samples with existing disease have also revealed a reduced risk of mortality, albeit small (2%), associated with higher levels of positive psychological well-being, independent of negative affect (Chida & Steptoe, 2008).

It is well-known that individuals with diabetes commonly experience depression, disease-related distress, and stress, all of which are linked to further comorbidity and mortality. Even in the presence of negative emotions such as depressive symptoms, however, our analyses revealed that positive affect was a protective factor for glycemic control in both the overall sample and the chronically ill, older group. Other studies have similarly found that the protective effects of positive emotions on health outcomes are independent from the adverse effects of these negative psychological factors. This finding makes sense given the fact that negative and positive affect are orthogonal constructs, such that the presence of positive affect, for instance, does not merely reflect the absence of negative affect (Massey et al., 2019). In fact, evidence suggests positive emotions can help buffer against depressive symptoms and stress, and their negative physiological consequences (Fredrickson & Levenson, 1998; Papousek et al., 2010; Tugade & Fredrickson, 2004), affording a mechanism by which experiencing more positive emotions leads to better glucose control.

PEWB may further improve glucose control by leading to the use of more effective coping strategies, including active coping, planning, positive reframing, and acceptance (Roesch & Weiner, 2001; McCoy & Theeke, 2019), rather than ineffective coping strategies such as smoking, alcohol use, and excessive eating. According to a recent meta-analysis of 22 primary studies, approach and problem-focused coping were associated with improved overall adjustment among persons with diabetes, and problem-focused coping moderated stress and negative emotions (Duangdao & Roesch, 2008). Surprisingly, emotion-based coping was also inversely related to depression and anxiety, and was associated with behaviors (e.g., medication adherence, blood sugar testing) that lead to positive diabetes outcomes (Duangdao & Roesch, 2008). In turn, these types of effective coping strategies have been inversely correlated with HbA1c (Shayeghian et al., 2015). In short, effective coping could serve as a behavioral mechanism by which PEWB leads to lower HbA1c (Mccoy & Theeke, 2019).

We found that BMI accounted for 53% of effect of positive affect on HbA1c in the overall sample, while exercise accounted for 27% of the effect of life satisfaction on HbA1c. Consistent with the former finding, some prior cross-sectional studies have reported that positive psychological wellbeing is associated with healthier weight status (Kelloniemi et al., 2005; Saloumi & Plourde, 2010; Robertson et al., 2015), although others have reported a negative (Li et al., 2018) or null (e.g., Sutin, 2013) association. Consistent with the latter finding, positive psychological wellbeing has been linked to more frequent exercise in several prior studies (Baruth et al., 2011; Kim et al., 2017; Lathia et al., 2017; Melin et al., 2003; Schwerdtfeger et al., 2010). The fact that PEWB is linked to and often precedes health behaviors such as exercise and the consumption of healthier diets comprised of fruits, vegetables, and whole grains (Lengyel et al., 2009; Yamasaki et al., 2007) suggests a third mechanism by which PEWB may help improve glucose control and protect against further morbidity amongst those with diabetes.

While there is limited literature on PEWB, diabetes, and blood sugar control, which we have integrated with our findings above, we could find almost nothing on life satisfaction and blood glucose control. It should be noted, however, that sometimes researchers include life satisfaction as part of the emotional well-being construct (Lopez et al., 2018). Given that life satisfaction was negatively related to HbA1c levels, our findings add this cognitive variable as potentially important in the relationship with blood glucose control.

### Limitations and future directions

Our study had several limitations that should be taken into account when interpreting the findings. Given the cross-sectional nature of this study, causality cannot be determined. Future studies should utilize longitudinal designs to more rigorously examine the directionality (including bidirectionality) and stability of the hypothesized pathways over time. It should also be noted that the PANAS, used to operationalize positive affect, is comprised of high arousal positive emotions (e.g., inspired, alert, determined, etc.), and does not include low arousal positive emotions such as feeling calm or peaceful, or other conventional positive emotions such as happy or joyful. This omission may limit the interpretation of our findings, as high and low arousal positive emotions may have different physiological and behavioral correlates. Although lower arousal positive affects have received less empirical attention, studies in the spiritual coping literature suggest that feelings of peace are associated with better glucose regulation (Gulbahar Eren et al., 2023). Relaxation interventions have also been linked to improved metabolic outcomes in individuals with diabetes (Yadav et al., 2021). Therefore, parasympathetic nervous system activity may contribute, as it supports glucose control and appears diminished in individuals with poor glycemic control (Hadad et al., 2022). Thus, more research is needed to ascertain whether our findings generalize to low arousal positive emotions. In addition, the chronic conditions were based on self-report. Furthermore, since we did not measure adherence to a diabetic regimen in this study, it would be beneficial for future studies to examine the role of adherence as a potential mediator of the relationship between positive affect and glucose control. There are fewer studies in the literature on life satisfaction and HbA1c, so this is another direction for future studies. In addition, future studies might examine the role of stress in this relationship. This paper investigated health behaviors as possible mediators, but other factors such as stress response pathways (Schneiderman et al., 2005) including HPA response, sympathetic activation, and gene expression may also play a role. Finally, positive coping strategies such as active coping, planning, positive reframing, and acceptance could also be explored to better understand the mechanisms at play in the relationship between PEWB and HbA1c.

### Clinical implications

PEWB factors may hold clinical utility for protecting and/or predicting blood sugar control and the development of hyperglycemia or diabetes. In the context of diabetes, it is important to note that the self-management burden of the disease is high, with patients required to adhere strictly to multiple health behaviors. Diabetes can be difficult to manage, particularly for older individuals. Future studies may determine whether PEWB interventions can help with both glycemic control and associated mental health issues and quality of life. If so, it may be worthwhile for health care providers to consider implementing intervention programs that incorporate improving PEWB of patients with diabetes (Rose et al., 2002). Meta-analyses show that PEWB interventions are effective in improving positive well-being (Kubzansky et al., 2023; Moskowitz et al., 2021), including among individuals with diabetes (Massey et al., 2019), although there are only a few studies in this population. However, the link between PEWB interventions and glycemic control needs further investigation, especially regarding physiological mechanisms, as most studies have examined the link between such interventions and behavioral self-management of diabetes, with unclear results (Pan & Yeung, 2023). Physicians may benefit from assessing patients’ PEWB to identify which individuals are at greater risk for worse health consequences. These assessments of PEWB require only short questionnaires with a few items, and could easily be incorporated into routine practice alongside the assessment of depression using the Patient Health Questionnaire-9.

PEWB has value in and of itself, and in its association with quality of life. Our study provides another reason to focus on PEWB, which is its association with better glycemic control. PEWB, consisting of positive affect and life satisfaction, is associated with HbA1c in both the general population and older, chronically ill individuals. This relationship holds even after controlling for depression. Health factors such as BMI and moderate exercise suggest pathways that mediate this relationship. Incorporating PEWB into a holistic assessment may be beneficial for physician patient management of glycemic control. Based on our cross-sectional data, future studies using longitudinal methods may investigate this relationship further and test whether PEWB has clinical utility in interventions designed to improve glycemic control in diverse populations.
